# Case report: prolonged durable clinical benefit and low toxicity from combination endocrine therapy in a patient with recurrent endometrial carcinoma

**DOI:** 10.3389/fonc.2023.1249370

**Published:** 2023-11-27

**Authors:** Joyce M. Cheng, Stephanie Gaillard, Anna L. Beavis, Tullia Rushton, Amanda N. Fader

**Affiliations:** ^1^ Johns Hopkins University School of Medicine, Baltimore, MD, United States; ^2^ Kelly Gynecologic Oncology Service, Department of Gynecology and Obstetrics, Johns Hopkins University School of Medicine, Baltimore, MD, United States; ^3^ Sidney Kimmel Cancer Center, Johns Hopkins University School of Medicine, Baltimore, MD, United States

**Keywords:** endometrial carcinoma, endometrioid, recurrence, endocrine therapy, tamoxifen, Progestational agent

## Abstract

**Background:**

Endometrial carcinoma is the most common gynecologic cancer, with increasing incidence and mortality. Combination endocrine therapy comprised of tamoxifen and progestational agents has demonstrated promising results in treating recurrent disease. This case report describes the prolonged clinical benefit of treatment with tamoxifen and megestrol acetate in a woman with recurrent, metastatic endometrial endometrioid carcinoma positive for estrogen (ER) and progesterone receptors (PR).

**Case:**

A 71-year-old gravida 1 para 1 woman presented with postmenopausal bleeding and vaginal discharge. Pelvic ultrasound and magnetic resonance imaging confirmed a 4.7 cm endometrial mass. The patient underwent a total laparoscopic hysterectomy, bilateral salpingo-oophorectomy, pelvic and para-aortic lymphadenectomy, and cystoscopy; pathology revealed a FIGO stage IA grade 1 ER/PR-positive endometroid endometrial adenocarcinoma. She continued under active surveillance for approximately 42 months until she experienced bone metastases in her pelvis, for which she received radiation therapy. Five months later, pulmonary metastases were detected, and she received six cycles of carboplatin and paclitaxel. She then started megestrol acetate and tamoxifen and has remained clinically stable with minimal side effects and reasonable quality of life for approximately 57 months.

**Conclusion:**

Our case suggests that combination endocrine therapy has the potential to provide substantial long-term clinical benefit in women with recurrent endometrial cancer and bone metastases, despite multiple prior treatments, allowing patients to experience stable disease and quality of life. In patients with recurrent endometrioid, ER/PR-positive disease, endocrine therapy alone or in combination with other targeted therapies are regimens that may be considered due to their low overall toxicity.

## Introduction

Endometrial carcinoma is the most common gynecologic cancer in the United States and the fourth most common malignancy in women, following breast, lung, and colorectal cancers (1). The incidence of endometrial carcinoma has been increasing, and is projected to surpass that of colorectal cancer by 2030 (1, 2). Likewise, the mortality rate has been increasing, despite 75% of women presenting with early-stage disease (1, 2). Recurrent disease, characterized by locoregional recurrence, distant metastasis, or both, occurs in approximately 7% to 15% of early-stage (I-II) patients (3). The prognosis for those with recurrent disease remains poor, with widely varying survival depending on the site of recurrence, tumor size, and treatment modality (4). For example, 36-month survival is significantly lower (8%) for women with a pelvic recurrence compared to an isolated vaginal recurrence (73%), and larger masses are associated with poorer local control (4, 5). In comparison, 36-month survival after distant relapse has been reported to be around 14% (5).

Treatment for recurrent endometrial carcinoma can include surgery, radiotherapy, chemotherapy, immunotherapy, endocrine treatment, or combinations of these modalities (3, 4). Endocrine therapy targets endometrial cancers positive for estrogen (ER) and progesterone receptors (PR) (3, 6). These hormone receptors, expressed in 90% of endometrial endometrioid carcinomas (EEC), have been associated with higher response rates to endocrine therapy (6). Based on current National Comprehensive Cancer Network (NCCN) guidelines, endocrine therapy is primarily used for small volume, low-grade tumors but may be used for recurrent and metastatic endometrial cancer (7). Endocrine therapy may include progestins, aromatase inhibitors, anti-estrogens, and gonadotropin-releasing hormone (GnRH) agonists (7).

Combination endocrine therapy comprised of tamoxifen and progestational agents is a promising treatment for advanced endometrial carcinoma. A phase II study (GOG 0153) by the Gynecologic Oncology Group (GOG) evaluated the efficacy of alternating megestrol acetate and tamoxifen in patients with recurrent or advanced endometrial carcinoma who had not received prior cytotoxic or endocrine treatment (8). This study found 38%, 24%, and 22% response rates in patients with histologic grade 1, grade 2, and grade 3 disease, respectively (8). Another GOG phase II study (GOG 0119) of tamoxifen combined with intermittent oral medroxyprogesterone acetate reported a response rate of 33% in patients with recurrent or metastatic endometrial carcinoma (9). Median progression-free survival (PFS) was three months, and median overall survival (OS) was 13 months (9). This case report describes prolonged clinical benefit from treatment with a combination of tamoxifen and megestrol acetate in a woman with ER/PR-positive, recurrent, metastatic EEC who was previously treated with surgery, radiation, and chemotherapy.

## Case report

The current patient provided consent for the write-up and publication of her clinical case. A 71-year-old white, non-Hispanic, gravida 1 para 1 woman presented with one month of postmenopausal bleeding and vaginal discharge. Her comorbidities included hypertension, hyperlipidemia, mitral valve prolapse, and gastroesophageal reflux disease. Her BMI has ranged between 22 to 24 kg/m^2^ throughout the course of her illness. The patient’s family history was negative for gynecologic, breast, or colon cancers. A pelvic ultrasound revealed a 3.3 cm hypervascular, hyperechoic endometrial mass. Subsequent magnetic resonance imaging (MRI) confirmed a 4.7 cm mass. An attempt at hysteroscopy, dilation, and curettage failed due to severe cervical stenosis and distorted cervical/uterine anatomy; thus, the patient underwent a robotic-assisted total laparoscopic hysterectomy, bilateral salpingo-oophorectomy, pelvic and para-aortic lymphadenectomy, and cystoscopy. Pathology revealed a 2009 FIGO staging system stage IA, grade 1 endometrioid endometrial adenocarcinoma with lower uterine segment involvement and 5/14 mm depth of invasion. The tumor was ER and PR positive. Adjuvant therapy was not recommended; instead, she continued under active surveillance. She had an Eastern Cooperative Oncology Group (ECOG) performance status (PS) of zero.

The patient experienced a 42-month disease-free survival until she complained of pelvic and hip discomfort. Computed tomography (CT) scan revealed bilateral pelvic masses: a dominant (12.4 x 10.3 x 15 cm) right-sided iliac fossa mass and a smaller (5.5 x 3.8 x 5.6 cm) left-sided lateral pelvic mass, both associated with bony destruction. Biopsy of the right pelvic mass confirmed osseous metastasis, indicating a recurrence of endometrioid adenocarcinoma with squamous differentiation, which was ER positive at 90%, PR positive at 40%, and PAX8 positive. The patient underwent pelvic radiation therapy (RT) dosed at 66 Gy in 30 fractions.

Five months later, restaging positron emission tomography (PET) CT demonstrated almost complete resolution of the patient’s pelvic disease with standardized uptake values (SUVs) of 4.1 and 2.2 for the right and left pelvic masses respectively, compared to 11.6 and 9.1 previously. PET/CT two months later reported the sizes of the right and left pelvic masses as 10.1 x 8.6 cm and 2.5 x 1.7 cm respectively, and a decrease in the maximum SUV of the large right-sided mass to 2.1. However, PET/CT also revealed a sub-centimeter upper right lobe pulmonary nodule. A subsequent CT scan two months later demonstrated four enlarging and/or new nodules in her right lung, measuring up to 6 mm, and slight enlargement of her left supraclavicular lymph node. Tumor mismatch repair (MMR) screening was proficient (i.e., MLH1, PMS2, MSH2, and MSH6 intact); thus, immunotherapy and genetic counseling were not recommended. At this point, her ECOG PS worsened to two due to chronic diarrhea and fatigue, likely associated with her prior RT.

The patient received six cycles of carboplatin (AUC 5) and paclitaxel (135 mg/m^2^/dose). Upon completion of chemotherapy, follow-up CT revealed stable pulmonary nodules and relatively unchanged partially calcified right iliac (9 x 7 cm) and left pelvic (2.0 x 1.2 cm) masses. The patient was then started on alternating megestrol acetate (80 mg) BID and tamoxifen (20 mg) BID every three weeks, based on her tumoral ER and PR positive status as indicated by her prior biopsy at the time of original recurrence. Zoledronic acid 4 mg IV every three weeks was also added. Concerns regarding side effects and tolerability were an additional reason for choosing endocrine therapy. After starting endocrine therapy, the patient experienced minimal side effects of nausea, emesis, and diarrhea, which quickly improved.

**Figure 1:**
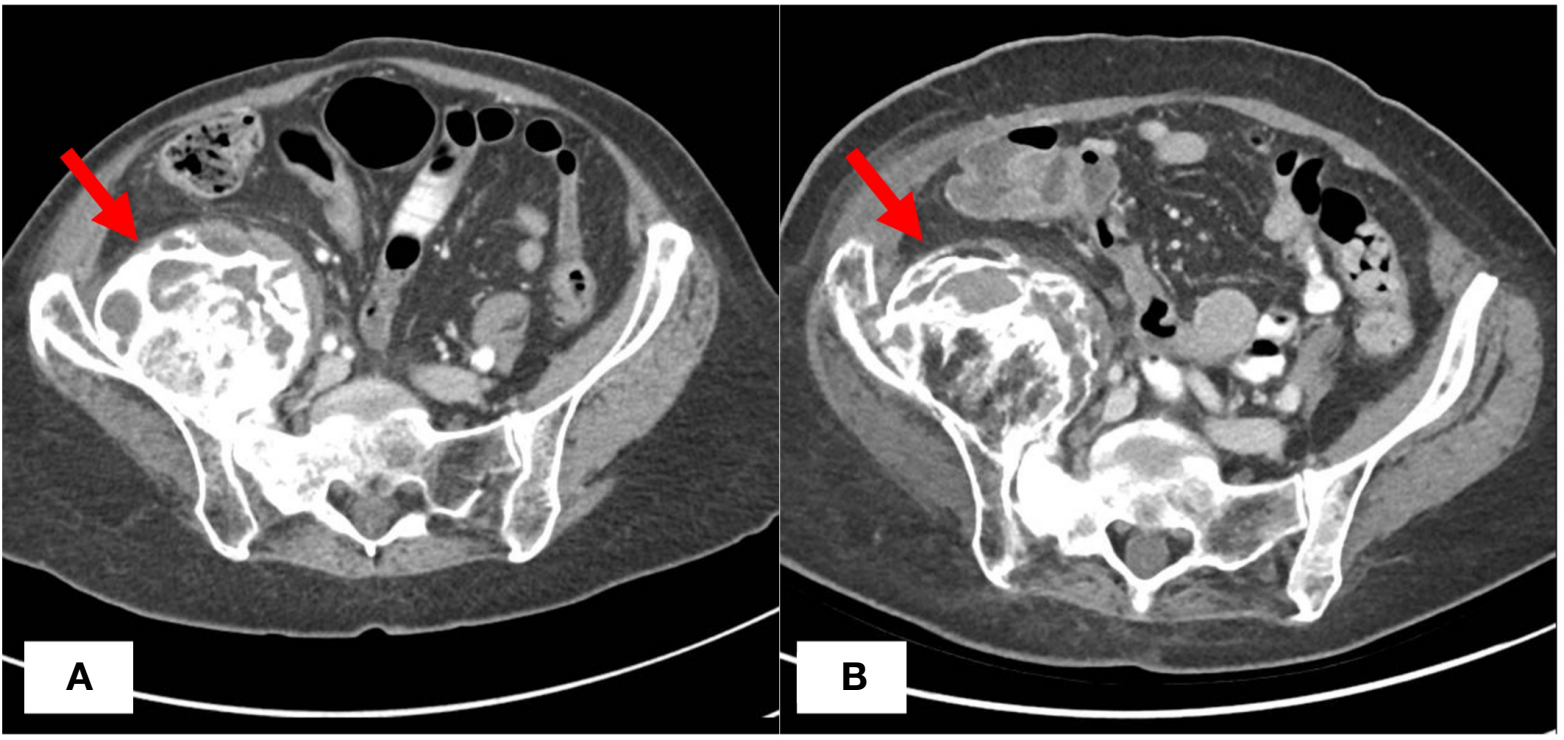
CT Imaging Before and After Treatment with Endocrine Therapy. CT abdomen/pelvis with IV contrast demonstrating largely unchanged right iliac mass before **(A)** and 57 months after **(B)** initiation of megestrol acetate and tamoxifen endocrine therapy.

Approximately 57 months later, the patient is still undergoing treatment with tamoxifen and megestrol acetate and her pelvic masses have remained stable on imaging (**Figure 1**). However, the patient continued to experience bilateral hip pain secondary to radiation-induced osteonecrosis and metastatic involvement of her right hip, which has been controlled with morphine. She subsequently developed an acetabular fracture due to radiation necrosis and the known expansile metastatic lesion’s involvement of the right acetabulum. The patient was referred to orthopedic oncology for a possible hip replacement but declined the procedure at that time. Her ECOG PS has remained stable. Since her cancer diagnosis 117 months ago and initiation of endocrine therapy 57 months ago, the patient has experienced relatively stable disease and is tolerating her treatment well.

## Discussion

In this report, we demonstrate durable clinical benefit lasting approximately 57 months from treatment with combination endocrine therapy of megestrol acetate and tamoxifen in a patient with recurrent endometrial cancer characterized by a large pelvic mass, bony metastases, and pulmonary nodules (**Table 1**). Notably, the patient had undergone prior conventional chemotherapy and pelvic radiation and was not deemed to be a good candidate for additional surgical or radiation treatment. This case demonstrates exceptional long-term disease control, given that the prognosis for widely metastatic, recurrent endometrial cancer is typically poor (4).

**Table 1:** Timeline of Events and Care for the Patient.

Timeline	Events
Month 0	• Presented with postmenopausal bleeding and vaginal discharge at age 61
Month 1	• Pelvic US and MRI revealed 4.7 cm mass
Month 2	• Underwent total laparoscopic hysterectomy, bilateral salpingo-oopherectomy, pelvic and para-aortic lymphadenectomy • Pathology revealed FIGO stage IA grade 1 ER/PR-positive endometroid endometrial adenocarcinoma • ECOG PS of 0 • Began active surveillance
Month 47	• CT revealed pelvic bony metastases • Began pelvic radiation therapy at 66 Gy in 30 fractions
Month 54	• PET/CT revealed pulmonary metastases
Month 56	• CT revealed enlarging pulmonary metastases and left supraclavicular lymph node • Underwent tumor MMR screening • Began 6 cycles of carboplatin (AUC 5) and paclitaxel (135 mg/m^2^/dose) • ECOG PS of 2
Month 60	• CT revealed stable pulmonary and pelvic disease • Began alternating megestrol acetate (80 mg) BID and tamoxifen (20 mg) BID
Month 117	• Continuing megestrol acetate and tamoxifen • Clinically stable at age 71 at the time of this report

US, ultrasound; MRI, magnetic resonance imaging; FIGO, International Federation of Gynecology and Obstetrics; ER, estrogen receptor; PR, progesterone receptor; ECOG, Eastern Cooperative Oncology Group; PS, performance status; CT, computed tomography; PET, position emission tomography; MMR, mismatch repair.

Combination endocrine therapy of tamoxifen and a progestational agent has improved outcomes for recurrent or advanced endometrial cancer. GOG studies have reported a median OS of up to 14 months (8, 9). Patients in these studies (GOG 0153 and GOG 0119) received endocrine therapy until disease progression or adverse events precluded further therapy (8, 9). Median PFS for those treated with megestrol acetate and tamoxifen was 2.7 months and median PFS for those treated with medroxyprogesterone acetate and tamoxifen was three months; however, considerably longer PFS was observed in our patient (8, 9). This may be partly because of the intensity of the tumoral hormone receptor expression, though this is not definitively known. In GOG 0119, four participants experienced extended survival of over 60 months since starting endocrine therapy; three had disease and one did not have any clinical or radiographic evidence of disease at the time of publication (9). Furthermore, in GOG 0119, 60% of all patients had received prior radiation therapy; 62% had pelvic disease, of which 68% received prior pelvic radiotherapy; and 18.3% had lung involvement, similar to our patient (9). Of the 58 patients with study drug exposure, 33% achieved a response, with 10% attaining a complete response and 22% attaining a partial response (9). It is believed that masses in previously irradiated fields are particularly challenging to treat due to the effects of radiation on the vasculature. In GOG 0153, 59% of patients had prior radiation therapy, 25% had pelvic disease, and 46% had lung involvement (8). Of the 56 patients studied, the overall response rate was 27%, with 21% achieving a complete response and 5% achieving a partial response (8). Although our patient did not attain a reduction in disease, she has achieved 57 months of stable disease and management of her cancer as a chronic condition, which is significant considering the usual poor prognosis for recurrent EEC with pelvic and lung involvement.

In our case, the patient maintained a reasonable quality of life throughout her treatment. Before her chemotherapy and endocrine therapy treatments, her ECOG PS declined from zero to two and remained at two while she underwent chemotherapy. Similar to GOG study participants receiving combination endocrine therapy, our patient mainly experienced low-grade toxicity, with chronic joint pain managed with analgesics and stable performance status (8, 9). In GOG 0153 and GOG 0119, weight changes were the most commonly reported adverse effect, followed by gastrointestinal effects (8, 9). Thromboembolic disorders were the most severe toxicity (8, 9). In our case, the patient has experienced minimal weight change and no thromboembolic disease. Endocrine therapy is a reasonable choice for select patients with endometrial cancer recurrence, resulting in fewer and less severe side effects than chemotherapy or radiation therapy. Thus, endocrine therapy may be a preferable option for long-term treatment to preserve patients’ quality of life.

More contemporary trials have explored the blockade of the PI3K/AKT/mTOR pathway as a method of overcoming resistance to endocrine therapy through synergistic antitumor effects. GOG 248 was a phase II trial that investigated temsirolimus with or without megestrol acetate and tamoxifen for endometrial cancer but found no significant difference between treatment groups and higher incidence of venous thrombosis in patients receiving all three medications (10). In contrast, a phase II study of everolimus and letrozole in patients with recurrent endometrial carcinoma demonstrated a clinical benefit rate (i.e., complete response, partial response, or stable disease) of 40% and a confirmed objective response rate (ORR) of 32% (11). Of the patients in this study, 94% received prior chemotherapy and 43% received prior radiotherapy (11). Most adverse events were low-grade toxicities (11). A more recent GOG phase II trial (GOG 3007) evaluating everolimus and letrozole (E/L) and medroxyprogesterone acetate and tamoxifen (M/T) in the treatment of advanced, persistent, or recurrent endometrial carcinoma found that both regimens demonstrated clinically meaningful efficacy (12). E/L demonstrated a response rate of 22%, clinical benefit in 78% of patients, and a median PFS of six months, while M/T demonstrated a response rate of 25%, clinical benefit in 69% of patients, and a median PFS of four months (12). Fifty-nine percent of the patients in each treatment arm received prior chemotherapy or chemoradiation (12). Notably, patients who had previously received chemotherapy had shorter median PFS than those without prior therapy (four months vs. 28 months for E/L and three months vs. five months for M/T, respectively) (12). However, our patient experienced extended clinical benefit, despite prior chemotherapy. The most commonly reported adverse event for both treatment arms in GOG 3007 was anemia, and more grade 3 or higher adverse events were reported for the E/L arm than the M/T arm (78% vs. 58%, respectively) (12). These studies highlight promising opportunities for treating recurrent endometrial cancer using endocrine therapy. Endocrine therapy may even be considered as an earlier line of treatment, possibly before RT or chemotherapy, given that response rates in GOG 3007 were strongest for patients who had not had prior chemotherapy (12).

Another therapeutic option for advanced or recurrent endometrial cancer is lenvatinib, a multikinase inhibitor, and pembrolizumab, a monoclonal antibody targeting programmed death protein 1. The Food and Drug Administration recently approved this treatment combination for endometrial carcinoma in July 2021; thus, our patient was not eligible for this therapy at the time of her treatment initiation. The KEYNOTE-146/Study 111 trial and the KEYNOTE-775/Study 309 trial both studied lenvatinib and pembrolizumab in patients with endometrial cancer and found increased median PFS and OS compared to those treated with chemotherapy (13, 14). Side effect profiles were similar, with grade 3 or higher adverse events reported in 66.9% of patients in KEYNOTE-146/Study 111, 88.9% of patients receiving lenvatinib and pembrolizumab in KEYNOTE-775/Study 309, and 72.7% of those receiving chemotherapy in KEYNOTE-775/Study 309 (13, 14). These trials present another alternative to chemotherapy for treating endometrial cancer.

Estrogen and progesterone receptor positivity has been associated with prognosis in endometrial cancer (15). A meta-analysis by Zhang et al. found that higher levels of ER/PR were associated with increased survival (15). However, there are currently no standard recommendations for endocrine therapy in endometrial cancer based on ER/PR status (16). In contrast, for breast cancer, the American Society of Clinical Oncology and the College of American Pathologists recommend ER/PR testing, which ultimately guides treatment decisions (17). The degree of ER/PR positivity needed to elicit a response to therapy for endometrial cancer is similarly not well defined. Various European oncology societies have advised a biopsy at the time of endometrial cancer recurrence to confirm hormone receptor status (16). Our patient was biopsied at recurrence to demonstrate her ER/PR positivity, and her ER/PR status may help account for the prolonged clinical benefit derived from her treatment. Progestational agents decrease PR expression, possibly hindering responses to treatment with endocrine therapy; however, adding tamoxifen, an estrogenic compound, may help restore this (18). Therefore, combination endocrine therapy may improve the duration and magnitude of treatment responses in patients with ER/PR-positive endometrial cancer (8, 9, 18). Future studies may be warranted to assess responses to combination endocrine therapy based on the degree of ER/PR positivity. In addition to her ER/PR positivity, our patient’s grade 1 disease and endometrioid histology may have improved her response to endocrine therapy (19, 20).

In summary, our case demonstrates that combination endocrine therapy has the potential to provide substantial long-term clinical benefit for women with recurrent, multi-site endometrial cancer with bone metastases, despite multiple prior treatments, allowing select patients to experience stable disease and quality of life. This case, as well as the studies presented, suggests that declines in performance status may often be attributed to adverse events from cancer treatment rather than from the tumor itself. In patients with recurrent endometrioid ER/PR-positive disease, endocrine therapy alone or in combination with other targeted therapies are regimens that may be considered due to their low overall toxicity.

## Data availability statement

The original contributions presented in the study are included in the article/supplementary material. Further inquiries can be directed to the corresponding authors.

## Ethics statement

Written informed consent was obtained from the participant/patient(s) for the publication of this case report.

## Author contributions

JC: Writing – Original Draft, Writing – Review & Editing. SG: Writing – Review & Editing. AB: Writing – Review & Editing. TR: Writing – Review & Editing. AF: Conceptualization, Writing – Review & Editing, Supervision. All authors contributed to the article and approved the submitted version.
